# Chemotherapy-Induced Monoamine Oxidase Expression in Prostate Carcinoma Functions as a Cytoprotective Resistance Enzyme and Associates with Clinical Outcomes

**DOI:** 10.1371/journal.pone.0104271

**Published:** 2014-09-08

**Authors:** Ryan R. Gordon, Mengchu Wu, Chung-Ying Huang, William P. Harris, Hong Gee Sim, Jared M. Lucas, Ilsa Coleman, Celestia S. Higano, Roman Gulati, Lawrence D. True, Robert Vessella, Paul H. Lange, Mark Garzotto, Tomasz M. Beer, Peter S. Nelson

**Affiliations:** 1 Divisions of Human Biology and Clinical Research, Fred Hutchinson Cancer Research Center, Seattle, Washington, United States of America; 2 Department of Medicine, Oregon Health and Sciences University, Portland, Oregon, United States of America; 3 Department of Medicine, University of Washington, Seattle, Washington, United States of America; 4 Department of Pathology, University of Washington, Seattle, Washington, United States of America; 5 Department of Urology, University of Washington, Seattle, Washington, United States of America; 6 Department of Urology and Cancer Institute, Oregon Health and Sciences University, Portland, Oregon, United States of America; 7 Section of Urology, Portland VA Medical Center, Portland, Oregon, United States of America; Boston University Goldman School of Dental Medicine, United States of America

## Abstract

To identify molecular alterations in prostate cancers associating with relapse following neoadjuvant chemotherapy and radical prostatectomy patients with high-risk localized prostate cancer were enrolled into a phase I-II clinical trial of neoadjuvant chemotherapy with docetaxel and mitoxantrone followed by prostatectomy. Pre-treatment prostate tissue was acquired by needle biopsy and post-treatment tissue was acquired by prostatectomy. Prostate cancer gene expression measurements were determined in 31 patients who completed 4 cycles of neoadjuvant chemotherapy. We identified 141 genes with significant transcript level alterations following chemotherapy that associated with subsequent biochemical relapse. This group included the transcript encoding monoamine oxidase A (MAOA). *In vitro*, cytotoxic chemotherapy induced the expression of MAOA and elevated MAOA levels enhanced cell survival following docetaxel exposure. MAOA activity increased the levels of reactive oxygen species and increased the expression and nuclear translocation of HIF1α. The suppression of MAOA activity using the irreversible inhibitor clorgyline augmented the apoptotic responses induced by docetaxel. In summary, we determined that the expression of MAOA is induced by exposure to cytotoxic chemotherapy, increases HIF1α, and contributes to docetaxel resistance. As MAOA inhibitors have been approved for human use, regimens combining MAOA inhibitors with docetaxel may improve clinical outcomes.

## Introduction

Despite numerous clinical trials conducted over a span of more than four decades, few interventions have extended survival in patients with advanced castration resistant prostate cancer (CRPC). Among these are the chemotherapeutic agent docetaxel which results in a median life-span extension of about 2 months, though few patients sustain durable complete remissions [1], [2]. For several types of solid tumors, notably neoplasms of the breast [3] and colon [4], cytotoxic drugs administered in conjunction with surgery or radiation have demonstrated survival benefits for those clinically-localized tumors with features indicating a predilection for early micrometastasis, or those with only documented local or regional spread. However, to date no studies have demonstrated a benefit for the addition of chemotherapy to primary surgical or radiotherapy approaches for prostate cancer as determined by outcomes of disease relapse or survival rates. Further, of five clinical studies evaluating neoadjuvant docetaxel alone or combined with other agents prior to prostatectomy, pathological complete responses are extremely rare [5]–[9]. These results indicate that prostate cancers either exhibit a high degree of intrinsic resistance to taxanes and other cytostatic and cytotoxic drugs, and/or rapidly acquire refractory phenotypes. Defining mechanisms underlying chemotherapy resistance is critical for selecting patients who may optimally benefit from specific regimens, and for designing new therapeutic strategies that either avoid – or specifically target resistance pathways.

To identify molecular changes associated with tumor cell exposure to chemotherapy agents commonly used in the treatment of prostate cancer, we conducted a prospective phase I-II clinical trial of neoadjuvant docetaxel and mitoxantrone in patients with high-risk localized prostate adenocarcinoma [10]. There were no complete pathological responses identified at the time of prostatectomy, and thus tumors analyzed after docetaxel and mitoxantrone exposure are presumably enriched for cells with molecular features contributing to therapy resistance. Although residual viable tumor cells were identified in each case, chemotherapy effects were evident [11]. We previously reported the results of profiling gene expression changes in microdissected tumors acquired in the context of this study and found that post-therapy gene expression varied substantially between individuals [12]. However, the short follow-up interval post-therapy precluded analyses to determine if gene expression changes were associated with tumor recurrence, a situation expected to occur through the survival and subsequent proliferation of subclinical micrometastasis present at the time of initial treatment.

In the present study we sought to test the hypothesis that gene expression changes in prostate cancer cells following exposure to cytotoxic chemotherapy would associate with clinical outcomes. The median follow-up of the cohort of prostate cancer patients treated with neoadjuvant docetaxel and mitoxantrone was 40 months at the time of this analysis. Molecular changes associating with relapse included increases in transcripts encoding monoamine oxidase A (MAOA). MAOA is a key enzyme involved in the degradation of the biogenic and dietary monoamine neurotransmitters such as 5-hydroxytryptamine (5-HT, or serotonin) and norepinephrine. Amine metabolism is linked to essential cellular processes such as cell growth and differentiation, and the catalytic byproducts of MAOA, such as hydrogen peroxide, are known to cause oxidative damage with implications for cancer, aging and neurodegenerative processes. We previously found that MAOA expression was upregulated in prostate cancers in association with higher Gleason grades [13], but mechanisms modulating cytotoxic drug effects have not been established. Two features of MAOA provided additional rationale for studies of this enzyme in the context of therapy resistance: approved inhibitors of MAOA are currently in routine clinical use for psychiatric illness and the safety and toxicity profiles are established. Second, MAOA enzymatic properties have been exploited to develop positron-emission tomography (PET)-based imaging reagents for localizing MAOA activity in humans. These features provide a clear path for the rapid clinical evaluation of MAOA as a therapeutic target in the context of advanced prostate cancer once sufficient preclinical evidence of roles in therapy resistance are established.

## Materials and Methods

### Ethics Statement

The clinical trial protocol (NCT00017563) was approved by the Institutional Review Boards of the Oregon Health & Science University, Portland VA Medical Center, Kaiser Permanente Northwest Region, Legacy Health System, and the University of Washington. All patients signed informed consent. All mouse studies were approved by the Fred Hutchinson Cancer Research Center (FHCRC) Institutional Animal Care and Use Committee (IACUC) and performed in accordance with the approved protocols.

### Patients and Study Description

Fifty-seven patients with high-risk localized prostate cancer (defined as TNM>cT2b or T3a or PSA≥15 ng/ml or Gleason grade ≥4+3) were recruited between 2001 and 2004 for a phase I-II clinical trial of neoadjuvant chemotherapy. The design of this clinical trial has been previously described [8], [10]. The trial identifier is NCT00017563.

### Prostate Tissue Collection, Processing and Microarray Profiling of Gene Expression

Details of the tissue collection and gene expression profiling have been previously described in detail [8]. Briefly, transrectal ultrasound-guided needle biopsies were obtained from each patient at study entry and snap-frozen in liquid nitrogen prior to chemotherapy. At radical prostatectomy, cancer-containing tissue samples were snap frozen immediately after prostate removal. Benign and neoplastic epithelial cells were separately acquired by laser-capture microdissection (LCM) using an Arcturus PixCell IIe microscope (Arcturus, Mountain View, CA). Total RNA was extracted from captured epithelium using a Picopure RNA isolation kit according to the manufacturer's instructions (Arcturus Inc., Mountain View, CA), and amplified using the messageAMP aRNA kit (Ambion, Austin, TX). Labeled cDNA probes were hybridized in a head-to-head fashion, pre-chemotherapy versus post-chemotherapy simultaneously to cDNA microarrays as we have previously described [14].

### Quantitative reverse transcription PCR

cDNA was synthesized from 1 µg amplified RNA (aRNA) from paired pre- vs. post-treated patient samples, or 1 µg RNA extracted from cultured cells, using 2 µg random hexamers for priming reverse transcription by SuperScript II (200 U per reaction; Invitrogen). Quantitative reverse transcription real-time PCR (qRT-PCR) reactions were done in duplicate, using approximately 5 ng of cDNA, 0.2 µM of each primer, and SYBR Green PCR master mix (Applied Biosystems, Foster City, CA) in a 20 µl reaction volume and analyzed using an Applied Biosystems 7700 sequence detector. Samples were normalized to the cycle threshold value obtained during the exponential amplification of GAPDH. Control reactions with RNA or water as template did not produce significant amplification products. The sequences of primers used in this study were: GAPDH forward, 5′-CCTCAACGACCACTTTGTCA-3′; GAPDH reverse, 5′-TTACTCCTTGGAGGCCATGT-3′; MAOA forward, 5′-AAAGTGGAGCGGCTACATGG-3′; MAOA reverse, 5′-CAGAAACAGAGGGCAGGTTCC-3′; pleiotrophin (PTN) forward: 5′-GGGCAGCAATTTAAATGTTATGACTA-3′; PTN reverse: 5′-ACCCCCATTTTGCTGACTACATT-3′.

### Cell Cultures and Treatments

The androgen responsive prostate cancer cell line, LNCaP and androgen insensitive prostate cancer cell line PC3 (both from the American Type Culture Collection, Manassas, VA) were grown in RPMI 1640 supplemented with 10% heat-inactivated fetal bovine serum and 100 IU/ml penicillin (Invitrogen Corp, Carlsbad, CA). The MAOA specific inhibitor, N-Methyl-N-propargyl-3-(2,4-dichlorophenoxy) propylamine hydrochloride (clorgyline), was purchased from Sigma-Aldrich (St Louis, MO). Docetaxel (provided by Sanofi-Aventis, Bridgewater, NJ) was diluted in 70% ethanol and used at 1 nM, 10 nM, and 100 nM concentrations for LNCaP cells and 50 nM and 200 nM for PC3 cells. For HIF1a expression studies, VCaP cells were grown in DMEM-F12 with 10% FBS. The MAOA specific inhibitor clorgyline (Sigma-Aldrich, St Louis, MO) was diluted in 70% ethanol and used at 1 µM concentration. Total RNA was isolated using the RNeasy kit (Qiagen, Valencia, CA). cDNA was synthesized using the SuperScript II Reverse Transcriptase kit (Invitrogen Corp, Carlsbad, CA). qRT-PCR reactions were done in triplicate, using SYBR Green master mix (Applied Biosystems, Foster City, CA) and analyzed using an Applied Biosystems 7700 sequence detector. Samples were normalized to the expression level of GAPDH.

### MAOA Expression

The full-length human MAOA cDNA was amplified from human placenta cDNA using the pair of primers, huMAOA_BstUI_170: GTCCGCGAAAGCATGGAG and huMAOA_EcoRI_1788: GCAGAGAGCATAAGAATTCAACTTCA. The amplification products were first cloned into pCR2.1 and then subcloned into the retro-viral vector pBABE-puro using BstU I and EcoR I sites. pBABE-puro-MAOA as well as the empty vector pBABE-puro were transfected into phoenix retro-viral packaging cells using Lipofectamine 2000 (Invitrogen Corp, Carlsbad, CA). The medium containing retrovirus expressing the vectors was collected and used to infect PC-3 cells followed by puromycin selection and confirmation of MAOA overexpression by both RT-PCR and western blot.

### Cell proliferation and apoptosis assays

Cell proliferation was assessed using the MTS dye reduction assay (Promega Corp, Madison, WI). Briefly, 10 µl of MTS reagent was added to each well of the 96-well plate and incubated at 37°C for 60 min. The color change was assessed by measuring the absorbance value of each well at 450 nm with an ELx808 BioTek absorbance microplate reader (BioTek Instruments, Winooski, VT). The Apo-One Caspase-3/7 assay (Promega Corp, Madison, WI) was used to assess apoptosis as per the manufacturer's instructions.

### MAO activity assay

MAO enzyme activity was assessed using the MAO-Glo assay (Promega Corp, Madison, WI). Cells were lysed using a passive lysis buffer (Promega Corp, Madison, WI) and a luminogenic MAOA substrate was added to the lysate to yield methyl ester luciferin. After incubating at room temperature for one hour, a detection reagent reconstituted from esterase and luciferase enzymes was added to each well and the luminescence was measured using a Berthold L9505 BioLumat microplate luminometer (Berthold Technologies, Oak Ridge, TN).

### Assay for Reactive Oxygen Species (ROS)

ROS was detected by CM-H2DCFDA (Invitrogen Corp, Carlsbad, CA). Cells were trypsinized and washed with PBS, incubated either with 10 µM of DCFDA in PBS or with PBS as a negative control at 37C for 30 min, washed with PBS and returned to media for a 30 min recovery period at 37C. Fluorescent cells in 10^5^ cells were counted by flow cytometry. Mean fluorescence intensity was used as a measurement of ROS. The assay was done in a triplicate.

### Western blot analysis

Whole cell lysates were prepared using RIPA buffer (25 mM Tris pH 7.6, 150 mM NaCl, 1% NP-40, 1% sodium deoxycholate, 0.1% SDS) with added protease inhibitors (Roche, Indianapolis, IN). Nuclear protein extracts were prepared using the Nuclear/Cytosol Fractionation Kit protocol (Biovision, Mountain View, CA). Thirty micrograms of protein were subjected to electrophoresis for 45 min at 200 V on a SDS polyacrylamide gel and transferred to a PVDF filter. The filters were blocked with 3% BSA for 1 h and then incubated overnight with anti-MAOA polyclonal antibody, anti- NFkB (Santa Cruz Biotechnology, Santa Cruz, CA), anti-HIF-1α (BD Biosciences, Franklin Lakes, NJ), or with anti-β-actin goat antibody (Promega Corp, Madison, WI). The filters were then incubated with species appropriate horseradish peroxidase labeled secondary antibodies (Thermo Scientific, Rockford, IL). Immuno blots were visualized using SuperSignal West Pico Chemiluminescent Substrate (Thermo Scientific, Rockford, IL).

### Xenograft experiments

Mouse xenograft models were generated via subcutaneous injections into the right flank of 8–10 week old male CB17 SCID mice (Taconic, Hudson, NY). Specifically, subcutaneous xenografts were derived utilizing either PC3 cells engineered to stably overexpress MAOA or empty vector controls. Prior to injection cells were grown to 90% confluence, washed once with PBS and resuspended in ice-cold PBS. Animals were anesthetized and 10^6^ cells were implanted subcutaneously in 200 microliters of 1∶1 PBS:Matrigel solution. Once tumors were established, mice were dosed with 5 mg/kg docetaxel weekly by IP injection. For both treatment arms (MAOA and Control) 12 xenografts were generated and tumor growth was monitored for a period of 4 weeks post injection at which point tumors were excised and snap frozen. Tumor measurements and animal weights were assessed three times weekly and weekly, respectively. Total tumor volumes were calculated with the following formula: (.5236)×(L_1_)×(L_2_)^2^, where L_1_ represents the long axis and L_2_ the short axis of the tumor [15]. Total RNA was extracted from frozen tumor sections using the RNeasy Mini RNA isolation kit (Qiagen, Valencia, CA) according to the manufactures protocol and further amplified to incorporate amino-allyl UTP using the MessageAmp II aRNA Kit (Ambion, Austin, TX). Samples were labeled and subsequently hybridized to Agilent 44K whole human genome expression arrays following the manufacturer's protocol (Agilent Technologies Inc., Santa Clara, CA). Data were processed using the Agilent Feature Extraction software, normalized, and filtered to remove probes with average intensity levels of <300. Resulting data were analyzed using the Statistical Analysis of Microarray (SAM) program [16], setting the significance threshold at a q-value of <0.5.

### Statistical analysis

In order to identify genes whose expression was significantly related to biochemical (PSA) relapse free survival, gene expression profiles were analyzed for associations with biochemical relapse (http://linus.nci.nih.gov/BRB-ArrayTools.html). Briefly, we computed a statistical significance level for each gene related to biochemical relapse-free survival based on univariate proportional hazards models. Significant gene lists were determined by a threshold of *p*<0.01. The association of expression change of MAOA and time to PSA relapse was analyzed using a Cox proportional hazards model (Stata 8.0, Texus). Multivariate models included age, clinical stage, pathology Gleason grade, baseline PSA level, and post-chemotherapy MAOA expression change. The final model only included factors meeting a significance threshold of *p*<0.1. The statistical significance of assays of MAOA activity, cell proliferation, and apoptosis was evaluated using a Student's t test for 2-group comparisons. A value of *p*<0.05 was considered to be significant. The Statistical Analysis of Microarray (SAM) program was used to analyze expression differences between control and MAOA transcript profiles using unpaired, two-sample t tests and controlled for multiple testing by estimation of q-values using the false discovery rate (FDR) method. To determine the association between MAOA and HIF1 changes following chemotherapy Pearson correlations were utilized. Finally, the statistical significance of data generated in the xenograft experiments were evaluated with the Proc GLM (General Linear Model) procedure in SAS (Cary, NC) utilizing a significance threshold of *p*<0.05.

## Results

### Clinical Trial Design and Patient Characteristics

**Figure 1:**
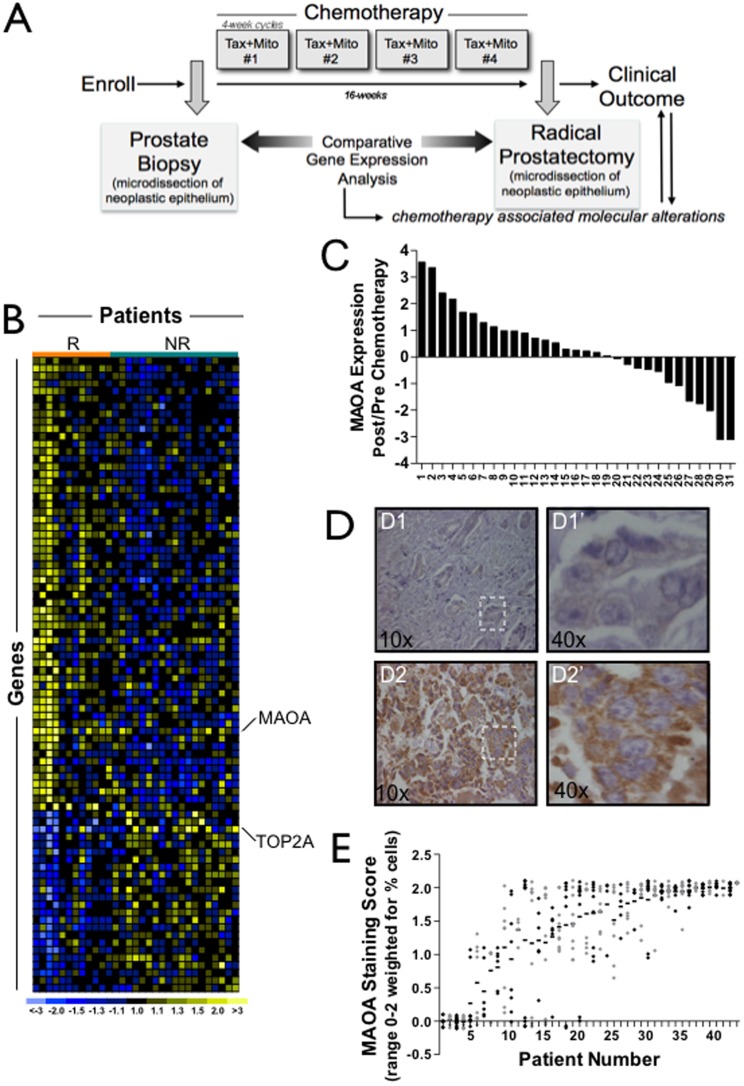
MAOA expression is induced *in vivo* following exposure to docetaxel and mitoxantrone. (A) Schema of the neoadjuvant chemotherapy and prostatectomy trial. (B) Heat-map of gene expression changes in prostate carcinoma cells that associate with biochemical relapse following radical prostatectomy. Columns are 31 patients and rows are 141 genes. Yellow indicates increased expression following chemotherapy. Blue indicates decreased expression and black indicates no change. Grey indicates absent or poor quality data. R is relapse and NR is no relapse. (C) MAOA transcript alterations shown for 31 study participants. Ratios are intra-individual post-treatment versus pre-treatment MAOA transcript abundance measurements determined by qRT-PCR from microdissected neoplastic epithelium. (D) MAOA protein expression determined by immunohistochemistry. Representative images of neoplastic prostate epithelium acquired before (D1) and after (D2) chemotherapy exposure. D1′ and D2′ indicate higher magnification images. Brown pigment indicates presence of MAOA protein. (E) MAOA protein expression by immunohistochemistry in prostate cancer metastasis from 44 patients. Each metastasis is indicated by a datapoint with multiple metastasis from the same individual located on the y-axis corresponding to each patient number. Grey and black datapoints alternate for ease of visualization. The horizontal line indicates the mean expression of MAOA across all metastasis for a given individual.

Patients with high-risk localized prostate cancer (N = 57) were enrolled in a neoadjuvant treatment protocol designed to administer four 28-day cycles of docetaxel 35 mg/m^2^ and escalating mitoxantrone doses (Phase I) to a maximum of 4 mg/m^2^ administered as 3 weekly doses followed by a 1-week off-treatment period (**Figure 1A**) [8]. Trans-rectal ultrasound-guided prostate biopsies were obtained prior to the first course of treatment. Within one month of completing chemotherapy each patient underwent a radical prostatectomy. We quantitated gene expression changes in benign and neoplastic prostate epithelium from 31 patients who completed the full courses of chemotherapy and for which sufficient tumor in the available pre- and post-therapy tissue samples permitted cell acquisition by microdissection. The attributes of the study participants have been described previously [8]. The median Gleason score was 7 and approximately half of the participants (16/31) had a clinical stage equal to or exceeding T3. No patient had a complete pathological response following chemotherapy. The median follow-up of the cohort was 40 months at the time of this analysis.

### Alterations in MAOA Expression Associate with Biochemical Relapse Following Neoadjuvant Chemotherapy and Prostatectomy

We measured gene expression changes in prostate cancer cells exposed to chemotherapy *in vivo* with the hypothesis that these molecular alterations would comprise resistance mechanisms and pathways that could be exploited as therapeutic targets to improve treatment responses. We used laser capture microdissection to acquire enriched populations of neoplastic epithelium from pre-treatment and post-treatment prostate tissue samples and quantitated gene expression changes following chemotherapy by microarray analysis [12]. At a median follow-up time of 40 months, using an intermediate end-point of serum PSA≥0.4 ng/ml and rising as a surrogate indicator of ultimate progression to metastasis [17], 11 out of 31 patients were determined to have biochemical progression. Of the chemotherapy-associated gene expression changes, 141 were significantly associated with PSA relapse-free survival (**Figure 1B** and **Table S1**). Several of these differentially-altered genes have previously been shown to influence chemotherapy resistance in other malignancies. For example, we found that down regulation of topoisomerase II alpha (TOP2A) associated with a higher rate of biochemical recurrence following chemotherapy. Low TOP2A levels have been associated with *in vitro* and *in vivo* resistance to chemotherapeutics including the TOP2A poison doxorubicin [18], [19] (**Figure 1B**).

Of those transcripts upregulated in neoplastic epithelial cells following chemotherapy (**Figure 1B**), we focused further on monoamine oxidase A (MAOA), as we previously found MAOA to be upregulated in localized prostate cancers, with higher expression in poorly-differentiated relative to well-differentiated tumors [13]. To confirm the microarray findings, we used qRT-PCR to assess MAOA transcripts in microdissected prostate cancers from the same patient before and after chemotherapy. In 18 of the 31 cases (58%) MAOA expression increased following treatment, and corresponding increases in MAOA protein levels were observed in three of three cases with elevated MAOA transcripts and sufficient tumor material in both pre- and post-treatment samples (**Figure 1C,D**).

We incorporated the magnitude of MAOA mRNA alterations in a univariate Cox Proportional Hazard Model to estimate hazard ratios of several risk factors including age, baseline serum PSA before chemotherapy, pathologic stage, and histological Gleason grade, using time to PSA relapse as the clinical outcome. Of these variables, only greater MAOA transcript change and higher Gleason grade were significantly associated with biochemical failure after chemotherapy and prostatectomy (**Table 1**). In order to measure the net effect of MAOA expression change associated with time to PSA relapse, we further fit MAOA expression change and prostatectomy Gleason grade into a multivariate Cox Proportional Hazard Model (**Table 2**). After adjusting for Gleason grade, the expression change of MAOA was marginally associated with time to PSA relapse (hazard ratio = 1.55, *p* = 0.068): the reduction in hazard ratio suggests that MAOA expression and status of tumor differentiation are associated.

**Table 1:** Univariate Model of MAOA Expression and Post-Therapy Biochemical Relapse.

Risk Factors	Hazard Ratio	*P*-value	95% CI
Gleason Grade*	2.02	0.014	1.15–3.56
Age	1.65	0.464	0.43–6.27
Baseline PSA	1.01	0.671	0.97–1.05
MAOA Expression Change * †	1.66	0.027	1.06–2.59

*p<0.05

† MAOA expression in pre-treatment and post-treatment samples was measured by a quantitative real-time PCR. Expression changes were measured by cycle threshold difference (▵CT) of MAOA between post-treatment and pre-treatment samples.

**Table 2:** Multivariate Model of MAOA Expression and Post-Therapy Biochemical Relapse.

Risk Factors	Hazard Ratio	*P*-value	95% CI
Gleason Grade	1.85	0.038	1.03–3.32
MAOA Expression Change ‡	1.55	0.068	0.97–2.47

‡ 0.05<*p*<0.1.

To assess whether MAOA may contribute to therapy resistance in disseminated tumor cells, we evaluated MAOA protein expression in prostate cancer metastasis acquired from 44 men with advanced castration resistant prostate cancer (**Figure 1E**). The majority of patients (90%) had at least one tumor with high MAOA expression (staining score ≥1), and concordant MAOA expression across multiple tumors (range 2–7 tumors per patient) from the same individual was evident.

### MAOA Activity is Altered by Chemotherapy and Influences Cell Proliferation *In Vitro*

**Figure 2:**
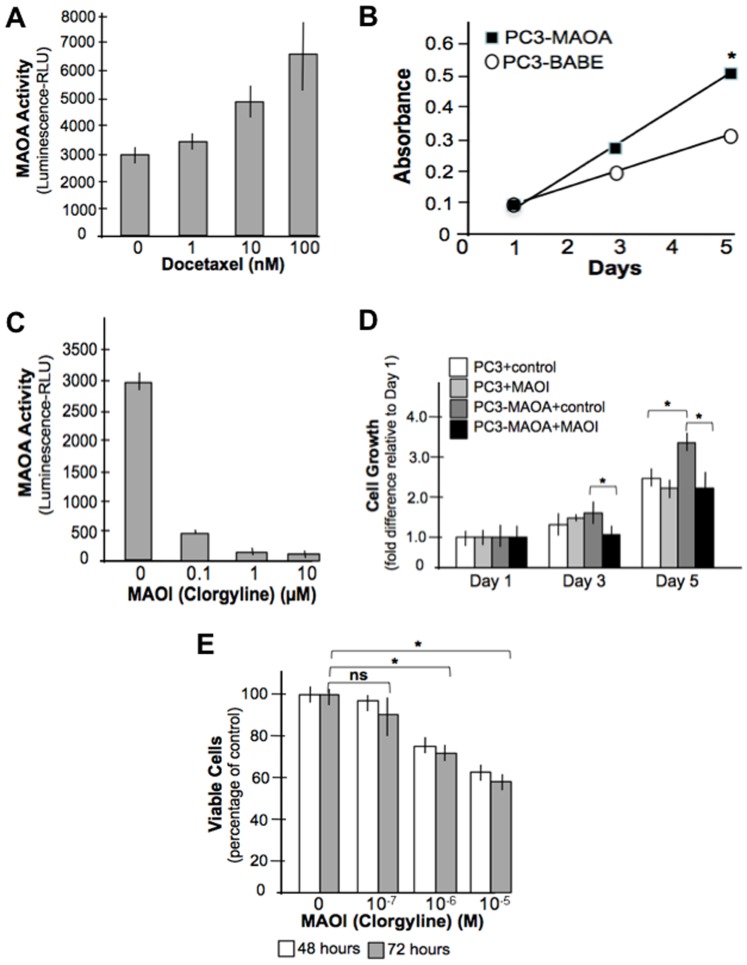
MAOA expression is induced by chemotherapy *in vitro* and promotes cell proliferation. (A) Treatment of LNCaP prostate cancer cells with docetaxel increases MAOA enzyme activity. (B) Over-expression of MAOA increases cell proliferation in PC3 prostate cancer cells. *p<0.05. (C) The irreversible MAOA inhibitor clorgyline reduces MAOA activity in LNCaP cells (*p<0.05). (D) Expression of MAOA in the PC3 prostate cancer cell line (PC3-MAOA) increases cell growth which is inhibited by clorgyline (MAOI). (E) LNCaP cell growth is inhibited by the MAOI clorgyline (*p<0.05).

To determine if chemotherapy directly influenced MAOA expression and to evaluate the influence of MAOA on cellular phenotypes, we measured steady-state MAOA expression in several androgen-sensitive and androgen-insensitive prostate epithelial cell lines. We found that androgen-responsive LNCaP cells expressed the greatest concentrations of MAOA transcripts and protein (**Figure S1A,B**). VCaP cells also expressed relatively high amounts of MAOA compared BPH-1 and 22RV1 prostate epithelial cells while androgen-insensitive DU145 and PC-3 showed limited, but detectable MAOA protein expression. None of the prostate cancer cell lines expressed appreciable levels of MAOB (**Figure S1A**). We treated the LNCaP prostate cancer cell line with increasing concentrations of docetaxel ranging from 1 to 100 nM. After 24 hours, MAOA activity increased significantly at each of the docetaxel concentrations, with higher levels affecting a greater magnitude of MAOA response (**Figure 2A**).

To determine if MAOA expression influences cell phenotypes, we expressed MAOA in PC3 cells, a line which exhibits very limited MAOA at baseline. Relative to vector controls, PC3 cells expressing MAOA increased growth by 30% after 5 days in culture (**Figure 2B**). Inhibitors of MAOA enzymatic activity have been developed, including several with approved clinical uses as anti-depressant agents. We determined that the MAOA inhibitor clorgyline could substantially reduce MAOA enzymatic activity in LNCaP cells at concentrations between 0.1–10 µM (**Figure 2C**). The MAOA-induced elevated growth rate in PC3 cells was eliminated by treatment with the MAOA inhibitor clorgyline at doses that effectively suppressed MAOA enzyme activity (**Figure 2D**). In control PC3 cells with absent MAOA expression at baseline, clorgyline had no effect on proliferation (**Figure 2D**). In contrast, clorgyline treatment of LNCaP (**Figure 2E**) and VCaP cells that normally express high steady-state levels of MAOA also resulted in significant growth suppression (*p*<0.05).

### Inhibition of MAOA Activity Modulates Docetaxel Effects on Prostate Cancer Cell Viability

**Figure 3:**
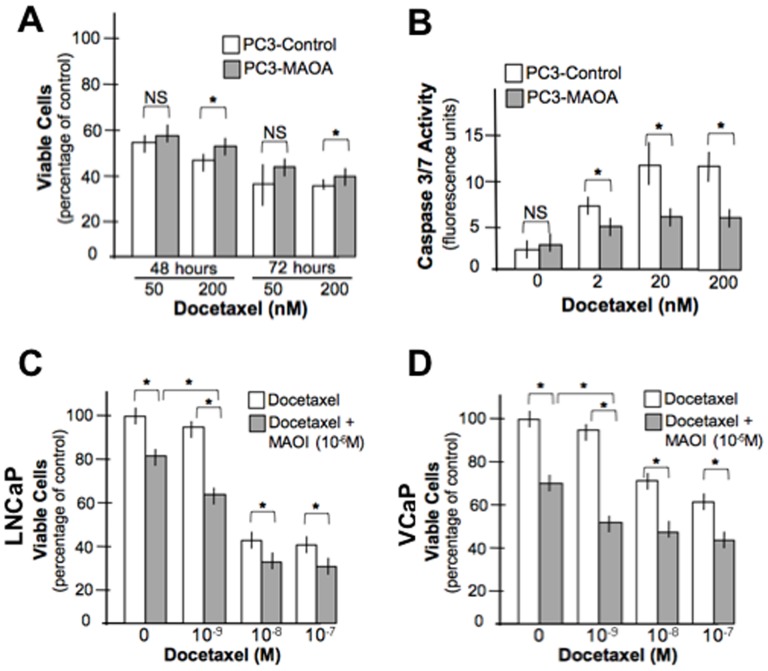
MAOA expression inhibits docetaxel cytotoxicity. (A) PC3 cells expressing MAOA exhibit enhanced cell survival following 48 and 72 hours of exposure to docetaxel (*p<0.05) (B) Elevated MAOA expression reduces cellular apoptosis following exposure to docetaxel. The addition of clorgyline enhances the cytotoxicity of docetaxel toward LNCaP (C) and VCaP (D) prostate cancer cells (*p<0.05).

In the neoadjuvant clinical trial of docetaxel and mitoxantrone (**Figure 1A**), increased MAOA expression following chemotherapy associated with biochemical relapse, suggesting MAOA may confer resistance to chemotherapy-induced cell death. In order to determine if inhibition of MAOA modifies chemoresistance, we compared the effects of docetaxel toward wild-type PC3 cells that express very low levels of MAOA, and PC3 cells engineered to express high levels of active MAOA enzyme. In short-term *in vitro* assays of cell viability, the expression of MAOA slightly increased the number of metabolically-active cells after 48 and 72 hours of treatment with 200 nM docetaxel; 8% and 5%, respectively (*p*<0.05) (**Figure 3A**). The expression of MAOA significantly reduced the cellular apoptotic response to docetaxel as determined by measurements of activated caspase 3 and 7 in the cells (*p*<0.05) (**Figure 3B**). We next treated LNCAP and VCaP cells expressing high endogenous levels of MAOA with the MAOA inhibitor clorgyline followed one hour later by different concentrations of docetaxel. In both cell lines, clorgyline substantially further reduced the number of viable cells resulting from docetaxel administration, with the most pronounced additive effects seen at lower concentrations of docetaxel (e.g. ∼30% further loss of cell viability at 10^−9^ M docetaxel; *p*<0.05) (**Figure 3C,D**).

### MAOA Expression Increases Reactive Oxygen Species and Activates Components of the HIF1A Program

**Figure 4:**
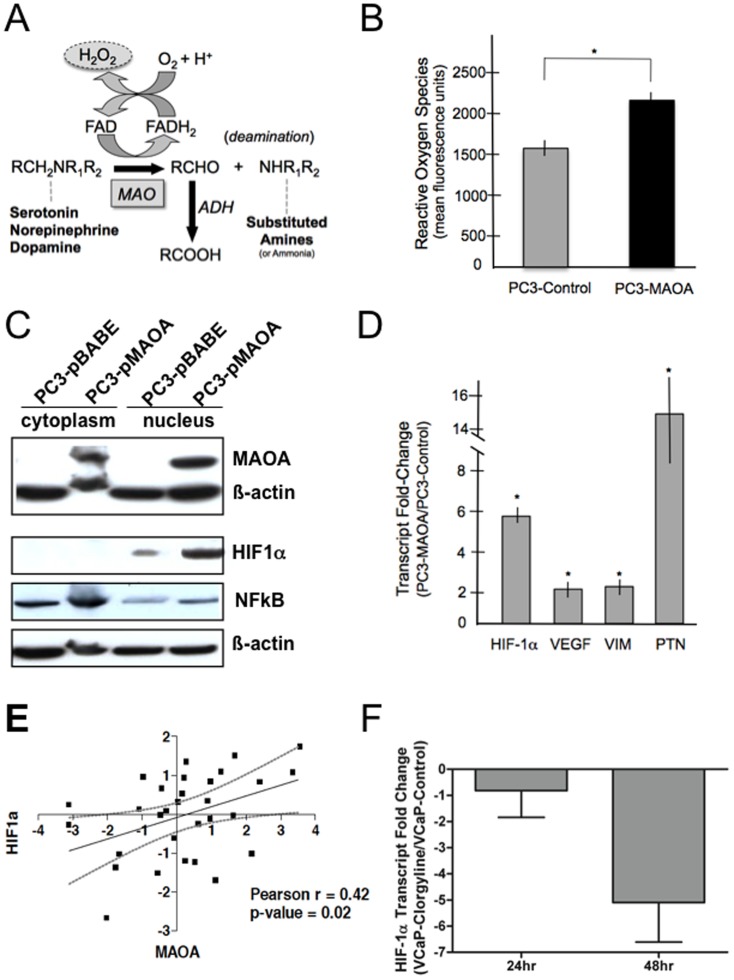
MAOA expression increases ROS and the expression of HIF1α and HIF1α pathway genes. (A) Deamination reaction catalyzed by monoamine oxidase (MAO) enzymes produces H_2_0_2_ as a reactive oxygen species (ROS) byproduct. (B) ROS levels are increased in PC3 cells expressing MAOA (*p<0.05). (C) Expression of MAOA in PC3 cells results in elevated nuclear HIF1α and NFκB protein. (D) Expression of MAOA in PC3 cells results in increased levels of transcripts encoding known HIF1A target genes. (E) Association of MAOA and HIF1 transcript level changes following chemotherapy. Plotted are the Log2 post-chemotherapy versus pre-chemotherapy transcript abundance ratios for each of 31 patients. The Pearson correlation value is 0.42 (*p* = 0.02). (F) Treatment of VCaP cells with the MAOA inhibitor clorgyline suppresses HIF1A expression. A four-fold reduction of HIF1A mRNA was quantitated by qRT-PCR at 48 hours relative to vehicle control (*p*<0.01).

We next sought to determine mechanisms by which MAOA could influence cellular resistance to chemotherapeutic agents. Monoamine oxidase enzymatic activity regulates oxidative deamination reactions of neurotransmitters such as serotonin, norepinephrine, and dopamine. Byproducts of this reaction include reactive oxygen species (ROS) such as H_2_0_2_ and hydroxyl radicals (**Figure 4A**). Using a fluorescence-based assay we determined that PC3 cells engineered to express MAOA had significantly higher levels of ROS than control PC3 cells propagated in identical growth medium and assayed at the same cell density (**Figure 4B**). ROS have been shown to activate several signaling programs including NFκB, MAP-kinase, and HIF1A. We determined that relative to control PC3 cells, PC3-MAOA cells expressed elevated levels of nuclear HIF1A (**Figure 4C**). Further, transcripts encoding the known HIF1A target gene, vascular endothelial growth factor (VEGF), and the EMT-associated proteins vimentin (VIM), and pleiotropin (PTN) [20]-[23] were increased in PC3 cells expressing high MAOA levels (**Figure 4D**). To further evaluate the clinical relevance of MAOA and HIF1A, we assessed whether elevated MAOA levels in prostate cancers treated with neoadjuvant chemotherapy associated with increased HIF1A *in vivo*. We found that MAOA expression following chemotherapy was significantly associated with HIF1A, r = 0.42 (*p* = 0.02) (**Figure 4E**). To further confirm that endogenous HIF1α expression was associated with MAOA activity, we treated high MAOA-expressing VCaP cells with clorgyline, and determined that after 48 hours of clorgyline exposure, HIF1A transcripts decreased more than 4-fold relative to cells treated only with vehicle control (**Figure 4F**).

### Elevated MAOA Expression Enhances Tumor Growth *In-vivo* and Alters Transcriptional Profiles

**Figure 5:**
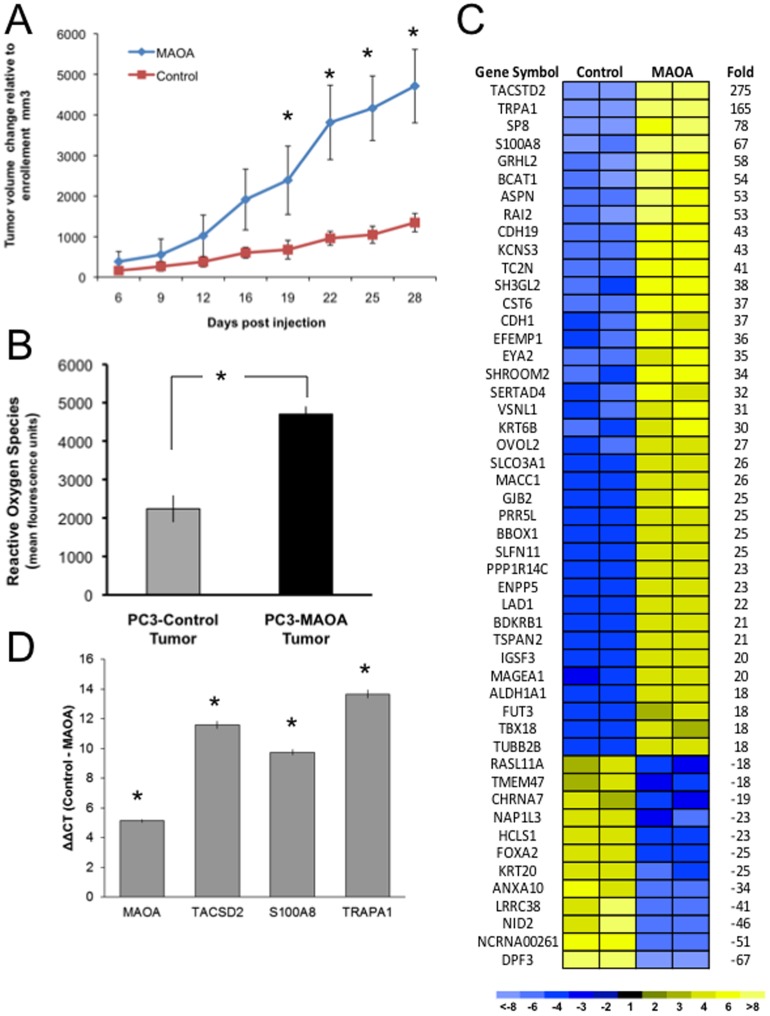
MAOA expression enhances *in-vivo* tumor growth and ROS production. (A) Animals harboring xenograft tumors overexpressing MAOA developed significantly larger tumor burdens over the four week observation period as compared to animals carrying the vector control tumors (*p<0.01 at the 28 week timepoint). (B) ROS levels are increased in xenograft tumor cells expressing MAOA (*p<0.05). (C) Heatmap of transcripts differentially expressed between PC3 vector control xenografts and PC3-MAOA expressing xenografts. Shown are transcripts increased (yellow) or decreased (blue) between the PC3-control versus PC3-MAOA tumors. (D) Quantitation of transcripts encoding MAOA, TACSD2, S100A8 and TRPA1 by qRT-PCR in PC3-MAOA versus PC3-control xenografts (*p<0.05).

To assess the influence of MAOA activity *in vivo*, we compared the growth of PC3 cells expressing either MAOA or empty vector controls. After implanting tumor cells into CB-17 SCID mice as xenografts, growth rates were monitored for a duration of four weeks. MAOA expression markedly influenced tumor growth with grafts of PC3 cells overexpressing MAOA averaging tumor volumes 5-fold greater than grafts comprised of control PC3 cells (*p*<0.01) (**Figure 5A**). Furthermore, similar to the *in vitro* cell culture studies, the xenograft tumors overexpressing MAOA had elevated levels of ROS compared to the control PC3 tumors (*p*<0.05) (**Figure 5B**).

Tumors collected for transcriptional analysis revealed a wide spectrum of gene expression differences between the MAOA and control xenografts, with over 1000 differentially expressed genes at a significance threshold of Q<0.1% including upregulation of HIF1α(2-fold) in the MAOA overexpressing tumors (**Figure 5C**). In addition, transcripts encoding several proteins known to be involved in oxygen sensing (TRPA1 [24]), ROS production (S100A8 [25]) and cellular proliferation (TACSTD2/TROP2 [26]; VSNL1 [27]) were substantially upregulated in tumors expressing MAOA (**Figure 5C and 5D**).

## Discussion

For reasons that remain unclear, adenocarcinomas arising in the prostate appear to be particularly resistant to the cytotoxic effects of commonly used anti-neoplastic drugs [1], [12], [28]. To identify resistance mechanisms, we designed a clinical trial to assess molecular features of tumor cells that associate with effects of chemotherapy exposure *in situ*. Among the gene expression changes we found to be altered by treatment, transcript levels of the gene encoding MAOA correlated with clinical relapse as defined by a rising PSA following radical prostatectomy. *In vitro* studies confirmed that docetaxel exposure increased the expression of MAOA in multiple prostate cancer cell lines, and inhibition of MAOA enzymatic activity using MAOA inhibitors enhanced the cytotoxicity of docetaxel.

Monoamine oxidase enzymes function to catalyze oxidative deamination reactions of neurotransmitters. Inhibition of MAO activity results in elevated levels of these amines in the central nervous system, a property responsible for antidepressant effects. Monoamine oxidase is encoded by two isozymes, MAOA and MAOB arranged in opposite orientation on the X chromosome, and expressed in the outer mitochondrial membrane in many diverse tissues throughout the body. MAOB preferentially metabolizes phenylethylamine and dopamine. MAOA metabolizes dopamine, serotonin and norepinephrine, though the dietary sympathomimetic tyramine is also a clinically-relevant substrate. Studies of MAO expression or activity in the context of cell growth, stress responses, apoptosis and neoplasia have primarily focused on cells derived from the central nervous system. Most of the available data indicate that biogenic amines serve as antiapoptotic factors and protect mitochondria against pro-apoptotic events by permitting closure of the mitochondrial permeability transition pore and preventing the initiation or propagation of the pro-apoptotic cascade [29]. In studies of neuronal cells, MAO inhibitors, leading to higher intracellular concentrations of monoamines and reduced production of reaction products such as H_2_0_2_, function to protect cells from pro-apoptotic stimuli [30]-[32]. A similar protective effect of MAO inhibitors toward melanoma cells has also been observed [29]. Further, studies of prostate cancer cells have demonstrated that serotonin, an MAO substrate, and serotonin receptor activity, associate with enhanced prostate cancer cell proliferation [33], [34].

In contrast to reports indicating that inhibiting MAO activity exerts effects that restrain tumor growth, several lines of evidence support a role for MAO in cancer promotion or progression. We previously found that MAOA expression correlated with prostate tumor cell differentiation status, with higher MAOA levels associated with higher Gleason patterns [13]. Subsequent studies demonstrated that MAOA inhibits prostate cell differentiation [35], and the MAOA inhibitor clorgyline is capable of suppressing pro-oncogenic programs in prostate cancer cells [36]. The expression of several other amine oxidases are increased in various cancer types such as those arising in the lung, breast, liver, and cervix [29]. Of interest, the administration of L-deprenyl, an inhibitor of MAOB, resulted in significant reductions in tumor incidence in a rodent model of carcinogen-induced breast cancer [37]. While a component of the anti-tumor effects were hypothesized to result from indirect activity toward prolactin production as well as neural-immune responses, direct effects of L-deprenyl on tumor cells were not ruled out.

An important area of inquiry centers on defining the mechanism(s) by which monoamine oxidases modulate tumor growth and chemotherapy resistance, and the corollary studies to determine how inhibitors of MAOA enhance chemotherapy sensitivity. The physiological functions of amine oxidases remain to be completely established, but known byproducts of amine metabolism include H_2_0_2_ and hydroxyl radicals that have the ability to promote MAP-kinase signaling through redox-sensitive pathways [29]. Our data indicate that one mechanism of therapy resistance links MAOA with the generation of ROS and activation of HIF1α. HIF1α expression – both protein stabilization and transcription, is promoted by elevated cellular ROS [38]. Several studies have demonstrated that elevated HIF1α enhances cell survival in the context of cytotoxic chemotherapy and radiation [39]-[41]. Thus, further investigations into MAOA-mediated therapy resistance mechanisms should seek to determine if the primary effects of MAOA inhibition on prostate cancer treatment responses operate through HIF1α versus other ROS-dependent or ROS-independent pathways.

Two additional attributes of MAOA provide further impetus for studies involving therapy for prostate carcinoma. First, tracers for positron emission tomography (PET) imaging of MAOA activity have been developed. For example, [11C] clorgyline and L-[11C] deprenyl have been used to image MAOA and MAOB, respectively, in studies of CNS enzyme activity [42]. The MAOA ligand Harmine has also been labeled with [11C] and used to image a subset of neuroendocrine carcinoid and pancreatic tumors [43]. These tracers offer the opportunity to quantitate the on-target effectiveness of inhibitors, as well as overall tumor responses. A second notable feature of MAO involves a recent finding that MAO blockade appears to exhibit differential cytoprotective effects toward benign versus malignant tissues [44]. Nontumorigenic and tumorigenic human cells were treated with MAOA and MAOB inhibitors, and exposed to gamma irradiation or cisplatin chemotherapy. The MAO inhibitors reduced radiation effects in the benign, but not the malignant cells, and MAO inhibition further suppressed the growth of malignant cells relative to those exposed only to radiation. MAO inhibitors also reduced cell death due to chemotherapy exposure in the benign but not malignant cells. Though the selective and irreversible MAOA inhibitor clorgyline is not available for clinical use due to adverse side-effect profiles, compounds that inhibit both MAOA and MAOB such as phenelzine and tranylcypromine, or reversible competitive inhibitors with excellent selectivity toward MAOA such as moclobemide, are approved for use as antidepressants in several countries. Exploiting their potential to enhance the effectiveness of therapies for prostate cancer could be evaluated rapidly.

## Supporting Information

Figure S1 A. Transcript levels of MAOA and MAOB in prostate cancer cell lines as determined by microarray hybridization. MAOB levels were at the lowest limit of detection across all cell lines. B. Western blot analysis of MAOA protein expression in prostate cancer cell lines. The detection of b-actin protein was used as a protein loading control. MAOA protein levels generally corresponded to the transcript levels across these lines through MAOA protein in PC-3 was detectable only with very prolonged exposures.
(TIFF)

Table S1 **Genes associated with biochemical relapse following neoadjuvant chemotherapy and radical prostatectomy.**
(PDF)
