# Supplementary figures and images for: Chemotherapy-Induced Monoamine Oxidase Expression in Prostate Carcinoma Functions as a Cytoprotective Resistance Enzyme and Associates with Clinical Outcomes
Source: PLoS One. 2014 Sep 8;9(9):e104271. doi: 10.1371/journal.pone.0104271 (PMC4157741; doi:10.1371/journal.pone.0104271)

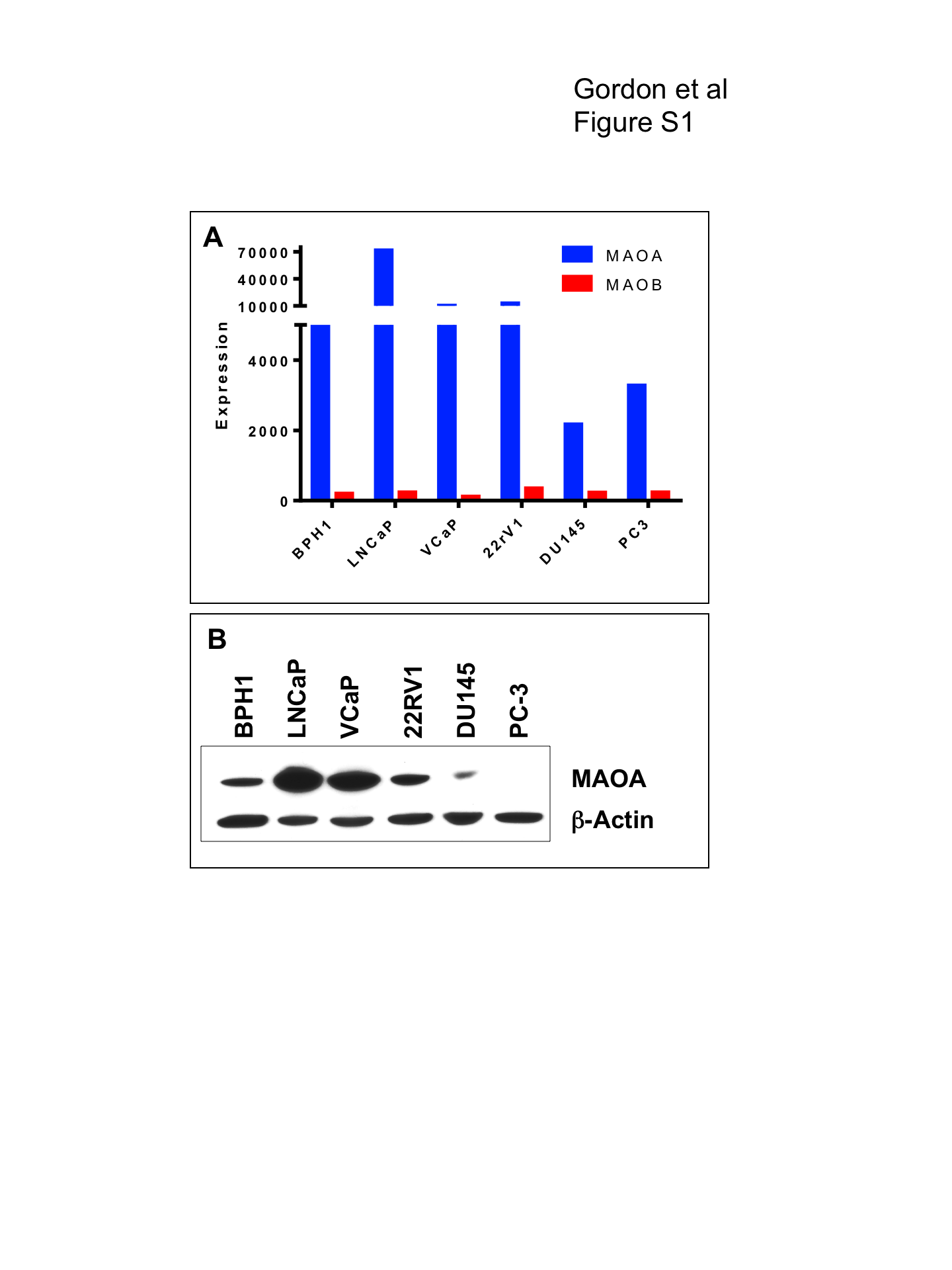

Supplement: Figure S1 — A. Transcript levels of MAOA and MAOB in prostate cancer cell lines as determined by microarray hybridization. MAOB levels were at the lowest limit of detection across all cell lines. B. Western blot analysis of MAOA protein expression in prostate cancer cell lines. The detection of b-actin protein was used as a protein loading control. MAOA protein levels generally corresponded to the transcript levels across these lines through MAOA protein in PC-3 was detectable only with very prolonged exposures. (TIFF) [file pone.0104271.s001.tiff]
