# Supplementary material for: Chemotherapy-Induced Monoamine Oxidase Expression in Prostate Carcinoma Functions as a Cytoprotective Resistance Enzyme and Associates with Clinical Outcomes
Source: PLoS One. 2014 Sep 8;9(9):e104271. doi: 10.1371/journal.pone.0104271 (PMC4157741; doi:10.1371/journal.pone.0104271)
Supplement: Table S1 — Genes associated with biochemical relapse following neoadjuvant chemotherapy and radical prostatectomy. (PDF) [file pone.0104271.s002.pdf]

Table S1: Genes associated with biochemical relapse following neoadjuvant chemotherapy and radical prostatectomy.

GenbankAccession	UniGeneID	GeneSymbol	GeneName	Parametric p-value	Hazard Ratio
AF012130	Hs.173984	TBX1	T-box 1	0.0039963	704.529
BC036014	Hs.253726	PAPOLA	Poly(A) polymerase alpha	0.0013303	132.59
BE046395	Hs.200716	MECP2	Methyl CpG binding protein 2 (Rett syndrome)	0.0034735	83.267
BC030520	Hs.549261	FLJ23790	Hypothetical protein FLJ23790	0.0003176	80.018
CD243774	Hs.532768	SERPINF1	Serine (or cysteine) proteinase inhibitor clade F (alpha-2 antiplasmin pigment epithelium derived factor) member 1	0.0055144	71.678
BG739892	Hs.279920	YWHAB	Tyrosine 3-monooxygenase/tryptophan 5-monooxygenase activation protein beta polypeptide	0.0027892	63.161
U79301	Hs.458285		Hypothetical LOC401131	0.0025675	48.787
BC014412	Hs.146668	TDE2	Tumor differentially expressed 2	0.0040079	44.452
AW272358	Hs.516633	NCKAP1	NCK-associated protein 1	0.0042037	43.603
AK129185			Mus musculus mRNA for mKIAA0666 protein	0.0012738	39.732
AI342442	Hs.513490	ALDOA	Aldolase A fructose-bisphosphate	0.0043196	37.154
BX379097	Hs.511425	SRP9	Signal recognition particle 9kDa	0.0011036	37.099
BG151231	Hs.437388	PIGT	Phosphatidylinositol glycan class T	0.0034315	35.442
AW675773	Hs.474643	HSPC117	Hypothetical protein HSPC117	0.0010067	31.328
BC006177	Hs.525629	MTA1	Metastasis associated 1	0.0096603	28.467
BC051800	Hs.491351	CLTC	Clathrin heavy polypeptide (Hc)	0.0009315	27.034
AK026668	Hs.127310	KIS	U2AF homology motif (UHM) kinase 1	0.0031015	26.334
CR625659	Hs.276315	FLJ21103	Hypothetical protein FLJ21103	0.000255	23.92
BQ049241	Hs.468442	CALM2	Calmodulin 2 (phosphorylase kinase delta)	0.0018956	23.418
CD251937	Hs.500104	AP3M1	Adaptor-related protein complex 3 mu 1 subunit	0.0027814	23.186
CR605573	Hs.339278	COPB	Coatomer protein complex subunit beta	0.0026692	21.829
BU153389	Hs.120196		Hypothetical LOC401057	0.0041886	21.602
BM511471	Hs.6551	ATP6AP1	ATPase H+ transporting lysosomal accessory protein 1	0.0053365	20.652
CR613279	Hs.444724	AZI2	5-azacytidine induced 2	0.0047856	20.528
AC119150			Homo sapiens chromosome 5 clone CTD-2280E9 complete sequence	0.002908	20.363
BQ004912	Hs.522394	HSPA5	Heat shock 70kDa protein 5 (glucose-regulated protein 78kDa)	0.0048385	19.152
BG117761	Hs.145710		Transcribed locus	0.008804	19.148
AV702705	Hs.545578	LOC153561	Hypothetical LOC389295	0.0088691	18.838
BU683394	Hs.511739	UBA2	SUMO-1 activating enzyme subunit 2	0.004473	17.603
BQ936969	Hs.503666	GPKOW	G patch domain and KOW motifs	0.0035106	17.416
BG031820	Hs.339639	COX7A2L	Cytochrome c oxidase subunit VIIa polypeptide 2 like	0.0020763	17.236
NM_006721	Hs.500118	ADK	Adenosine kinase	0.0035835	16.902
NM_024660	Hs.352548	FLJ22573	Hypothetical protein FLJ22573	0.0061417	16.589
AF229253	Hs.435771	API5	Apoptosis inhibitor 5	0.0093554	15.474
BC012540	Hs.459691	PDPK1	3-phosphoinositide dependent protein kinase-1	0.002693	15.187
BC033702	Hs.22137		Similar to NAD-dependent deacetylase sirtuin 5 (SIR2-like protein 5)	0.0033904	15.094
BM786880	Hs.503709	PORIMIN	Pro-oncosis receptor inducing membrane injury gene	0.000719	14.845
CA502894	Hs.12102	SNX3	Sorting nexin 3	0.002654	14.672
BG748148	Hs.71787	MRPS7	Mitochondrial ribosomal protein S7	0.0070516	14.541
AW338693	Hs.377155	LYRIC	LYRIC/3D3	0.0006445	14.508
AL832398	Hs.518099	MGC26717	Hypothetical protein MGC26717	0.006282	14.279
BG281999	Hs.533712	RBM4	RNA binding motif protein 4	0.0029559	13.904
AI262970	Hs.145586	COL4A6	Collagen type IV alpha 6	0.0040389	13.076
BC071976	Hs.432548	C10orf18	Chromosome 10 open reading frame 18	0.0026494	12.931
BC002775	Hs.462035	UBE2G1	Ubiquitin-conjugating enzyme E2G 1 (UBC7 homolog C. elegans)	0.005449	11.445
AW468450	Hs.462492	USP22	Ubiquitin specific protease 22	0.0010451	11.31
BT009841	Hs.134822	SNX2	Sorting nexin 2	0.0078492	11.016
CF126878	Hs.472558	SDBCAG84	Serologically defined breast cancer antigen 84	0.0092494	10.656
AC009948			Homo sapiens BAC clone RP11-65L3 from 2 complete sequence	0.0051814	10.559
BU553804	Hs.321231	B4GALT3	UDP-Gal:betaGlcNAc beta 14- galactosyltransferase polypeptide 3	0.0099664	10.556
BU608264	Hs.433201	CDK2AP1	CDK2-associated protein 1	0.0067662	9.839
AK074131	Hs.237056	SH3MD4	SH3 multiple domains 4	0.0069578	9.659
AI589417	Hs.183684	EIF4G2	Eukaryotic translation initiation factor 4 gamma 2	0.0029559	9.481
BP210312	Hs.293736	ADNP	Activity-dependent neuroprotector	0.0095043	9.301
BQ898571	Hs.446123	CAPZA2	Capping protein (actin filament) muscle Z-line alpha 2	0.0096738	9.152
BQ646781	Hs.173611	NDUFS2	NADH dehydrogenase (ubiquinone) Fe-S protein 2 49kDa (NADH-coenzyme Q reductase)	0.0056338	9.115
CR598326	Hs.2853	PCBP1	Poly(rC) binding protein 1	0.0048949	9.047
BQ223305	Hs.447492	PGAM1	Phosphoglycerate mutase 1 (brain)	0.0028214	8.766
AW473264	Hs.310645	RAB1A	RAB1A member RAS oncogene family	0.0018301	8.715
CF127525	Hs.516808	PDE6D	Phosphodiesterase 6D cGMP-specific rod delta	0.007736	8.697
AY598333	Hs.463936	DKFZP586L0724	DKFZP586L0724 protein	0.0077964	8.629
CR591073	Hs.173611	NDUFS2	NADH dehydrogenase (ubiquinone) Fe-S protein 2 49kDa (NADH-coenzyme Q reductase)	0.0054124	8.616
BC030205	Hs.437322	TNFAIP6	Tumor necrosis factor alpha-induced protein 6	0.0055896	8.614
BU686978	Hs.519276	MAPKAPK2	Mitogen-activated protein kinase-activated protein kinase 2	0.0080558	8.604
NM_001006115	Hs.549093	IHPK1	Inositol hexaphosphate kinase 1	0.0055459	8.549
BX402450	Hs.523512	TSG101	Tumor susceptibility gene 101	0.00718	8.141
BQ929330	Hs.512636	PNRC2	Proline-rich nuclear receptor coactivator 2	0.0050604	7.845
CN276618	Hs.492407	YWHAZ	Tyrosine 3-monooxygenase/tryptophan 5-monooxygenase activation protein zeta polypeptide	0.0010189	7.768
AL519777	Hs.476365	SCP2	Sterol carrier protein 2	0.0090288	7.61
AA570078	Hs.509736	HSPCB	Heat shock 90kDa protein 1 beta	0.0056374	7.579
AK096496	Hs.525299	BRMS1L	Breast cancer metastasis-suppressor 1-like	0.0087847	7.506
BU858760	Hs.238990	CDKN1B	Cyclin-dependent kinase inhibitor 1B (p27 Kip1)	0.0061688	7.253
BU197372	Hs.54649	RY1	Putative nucleic acid binding protein RY-1	0.0074653	7.017
NM_001759	Hs.376071	CCND2	Cyclin D2	0.0041168	6.854
CD513632	Hs.20107	KNS2	Kinesin 2 60/70kDa	0.0013706	6.692
BQ691926	Hs.499839	RPL7A	Ribosomal protein L7a	0.0090379	6.633
CK299653	Hs.498792		Full-length cDNA clone CS0DF038YM01 of Fetal brain of Homo sapiens (human)	0.0031383	6.508
U72391	Hs.388613	NEO1	Neogenin homolog 1 (chicken)	0.0027666	6.506
AB020661	Hs.377090	ZHX2	Zinc fingers and homeoboxes 2	0.0035153	6.44
CR607207	Hs.148330	ARF4	ADP-ribosylation factor 4	0.0021941	6.43
BQ213225	Hs.223894	IL12RB1	Interleukin 12 receptor beta 1	0.0087456	6.399
M33647	Hs.408528	RB1	Retinoblastoma 1 (including osteosarcoma)	0.0067608	6.327
CR740528	Hs.435850	LYPLA1	Lysophospholipase I	0.0092399	5.939
AL551352	Hs.271643	C6orf209	Chromosome 6 open reading frame 209	0.0061113	5.715
CR599220	Hs.522932	NCOA4	Nuclear receptor coactivator 4	0.0041822	5.647
NM_002906	Hs.263671	RDX	Radixin	0.0026572	5.58
BQ879737	Hs.430425	GNB1	Guanine nucleotide binding protein (G protein) beta polypeptide 1	0.0036276	5.52
NM_014673	Hs.232002	KIAA0103	KIAA0103	0.0051335	5.481
BF970934	Hs.13313	CREBL2	CAMP responsive element binding protein-like 2	0.00254	5.378
S79871	Hs.67397	HOXA1	Homeo box A1	0.0029612	5.332
AK098027	Hs.470627	LCK	Lymphocyte-specific protein tyrosine kinase	0.0055352	5.225
CR599482	Hs.74137	TMP21	Transmembrane trafficking protein	0.00071	5.174
BE301432	Hs.445203	DNAJA1	DnaJ (Hsp40) homolog subfamily A member 1	0.0018501	5.105
BQ437223	Hs.381219	RPL15	Ribosomal protein L15	0.0050586	5.082
BC068486	Hs.503222	RAB6A	RAB6A member RAS oncogene family	0.0034234	4.74
BC063665	Hs.374257	SIAT4A	Sialyltransferase 4A (beta-galactoside alpha-23-sialyltransferase)	0.0093538	4.719

AF130048	Hs.440643	LOC286144	Hypothetical protein LOC286144	0.0094495	4.684
AK129885	Hs.180414	HSPA8	Heat shock 70kDa protein 8	0.0054466	4.521
NM_152876	Hs.244139	TNFRSF6	Tumor necrosis factor receptor superfamily member 6	0.0069947	3.248
NM_002954	Hs.546292	RPS27A	Ribosomal protein S27a	0.0098809	2.706
BC008064	Hs.183109	MAOA	Monoamine oxidase A	0.0072309	2.563
AW172827	Hs.156346	TOP2A	Topoisomerase (DNA) II alpha 170kDa	0.0015367	0.395
AI819354	Hs.529735	AADAT	Aminoadipate aminotransferase	0.0092118	0.321
BX110993	Hs.539017		Transcribed locus	0.0074076	0.306
CN343714	Hs.520287	C6orf111	Chromosome 6 open reading frame 111	0.0097785	0.283
AA447274	Hs.171299	ZBTB16	Zinc finger and BTB domain containing 16	0.008577	0.274
AP001137			Homo sapiens genomic DNA chromosome 21q21.1-q21.2 LL56-APP region clone:B812P3	0.0059564	0.251
BC000133	Hs.388024	ZNF76	Zinc finger protein 76 (expressed in testis)	0.0073698	0.212
CN417121	Hs.461412		mitochondria: CDNA clone IMAGE:5268125 partial cds	0.0092257	0.208
AC027332			Homo sapiens chromosome 5 clone CTD-2193I13 complete sequence	0.0089469	0.193
AC080089			Homo sapiens BAC clone RP11-785J10 from 4 complete sequence	0.0091001	0.193
AA447275	Hs.461453		Similar to 40S ribosomal protein S3	0.0068595	0.187
BU957055	Hs.34180	UGCGL1	UDP-glucose ceramide glucosyltransferase-like 1	0.0060436	0.182
AC096713			Homo sapiens BAC clone RP11-1J11 from 4 complete sequence	0.0038146	0.173
BC028103	Hs.43627	SOX12	SRY (sex determining region Y)-box 12	0.0080058	0.166
AL553289	Hs.517293	F11R	F11 receptor	0.0091756	0.162
BC004937	Hs.376064	NOL5A	Nucleolar protein 5A (56kDa with KKE/D repeat)	0.0081596	0.155
AI914440	Hs.530588	SIAT8F	Sialyltransferase 8F (alpha-2 8-sialyltransferase)	0.0067422	0.148
BC003566	Hs.514802	ZNF24	Zinc finger protein 24 (KOX 17)	0.0081861	0.13
AK025129	Hs.158783	FLJ20618	Hypothetical protein FLJ20618	0.0055129	0.129
AC090214			Homo sapiens chromosome 18 clone RP11-800G9 complete sequence	0.0032736	0.127
BI753585	Hs.530084	PILRB	Paired immunoglobin-like type 2 receptor beta	0.0025035	0.116
CR619052	Hs.222510	DAZAP1	DAZ associated protein 1	0.0060142	0.108
AB029020	Hs.480597	USP33	Ubiquitin specific protease 33	0.0056552	0.106
CR621627	Hs.549260	WBSCR20B	Williams-Beuren Syndrome critical region protein 20 copy B	0.005646	0.097
BC008208	Hs.8765	DDX42	DEAD (Asp-Glu-Ala-Asp) box polypeptide 42	0.0024553	0.096
BM850369	Hs.356794	RPS24	Ribosomal protein S24	0.0038894	0.093
BP371798	Hs.491494	CCT3	Chaperonin containing TCP1 subunit 3 (gamma)	0.0033583	0.087
AK055516	Hs.444934	NRD1	Nardilysin (N-arginine dibasic convertase)	0.0009029	0.083
BC008909	Hs.511817	PHIP	Pleckstrin homology domain interacting protein	0.0066185	0.077
AB040911	Hs.505469	RACGAP1	Rac GTPase activating protein 1	0.0090509	0.076
AK024755	Hs.398178	C4orf9	Chromosome 4 open reading frame 9	0.0037649	0.074
AF002999	Hs.63335	TERF2	Telomeric repeat binding factor 2	0.007494	0.071
M15990	Hs.194148	YES1	V-yes-1 Yamaguchi sarcoma viral oncogene homolog 1	0.0030967	0.056
NM_207047	Hs.511916	ENSA	Endosulfine alpha	0.0093066	0.049
NM_033527	Hs.515679	CDC2L2	Cell division cycle 2-like 2 (PITSLRE proteins)	0.0045188	0.048
CR596896	Hs.14839	POLR2G	Polymerase (RNA) II (DNA directed) polypeptide G	0.0015507	0.047
AJ420461	Hs.144073	QSCN6L1	Quiescin Q6-like 1	0.0088981	0.045
AL133562	Hs.464391	TBCD	Tubulin-specific chaperone d	0.000816	0.042
CN280396	Hs.90436	SPAG7	Sperm associated antigen 7	0.0040991	0.039
CN409645	Hs.438236	ABLIM1	Actin binding LIM protein 1	0.0086367	0.008
